# Exploring miRNA Research in Colorectal Cancer: Insights from a Bibliometric Analysis

**DOI:** 10.3390/pharmaceutics17081084

**Published:** 2025-08-21

**Authors:** Emanuele Piccinno, Michelangelo Aloisio, Viviana Scalavino, Francesco Russo, Gianluigi Giannelli, Davide Guido, Grazia Serino

**Affiliations:** 1 Laboratory of Molecular Medicine, National Institute of Gastroenterology IRCCS “S. de Bellis”, Via Turi 27, Castellana Grotte, 70013 Bari, Italy; emanuele.piccinno@irccsdebellis.it (E.P.); viviana.scalavino@irccsdebellis.it (V.S.); 2 Functional Gastrointestinal Disorders Research Group, National Institute of Gastroenterology IRCCS “S. de Bellis”, Via Turi 27, Castellana Grotte, 70013 Bari, Italy; michelangelo.aloisio@irccsdebellis.it (M.A.); francesco.russo@irccsdebellis.it (F.R.); 3 Scientific Direction, National Institute of Gastroenterology IRCCS “S. de Bellis”, Via Turi 27, Castellana Grotte, 70013 Bari, Italy; gianluigi.giannelli@irccsdebellis.it; 4 Data Science Unit, National Institute of Gastroenterology IRCCS “S. de Bellis”, Via Turi 27, Castellana Grotte, 70013 Bari, Italy

**Keywords:** bibliometric analysis, biomarkers, colorectal cancer, microRNA, therapeutic targets, tumorigenesis

## Abstract

**Background/Objectives**: Despite advances in diagnosis and treatment, colorectal cancer (CRC) remains one of the most prevalent and challenging malignancies worldwide. The dysregulation of microRNAs (miRNAs) has emerged as a critical factor in CRC onset, progression, and therapeutic resistance. This study aims to provide an overview of global research trends on miRNAs in CRC, (i) identifying the most studied miRNAs, (ii) exploring under-investigated areas, and (iii) highlighting emerging themes and potential future directions. **Methods**: To assess the evolution of the global miRNA–CRC research trends, we conducted a bibliometric analysis of 828 CRC–miRNA-focused articles published between 2008 and 2024, sourced from the Scopus database. Bibliometric mapping was performed using the R/Bibliometrix package and by leveraging a customized Python-based pipeline, which is useful for extracting and validating miRNA identifiers (miRNA IDs) based on the miRBase database. This miRNA ID-related approach enabled us to systematically identify the most frequently studied miRNAs over time while highlighting underexplored miRNA. **Results**: The analysis revealed a substantial and accelerating publication growth rate, delineating three major phases in CRC–miRNA research. China emerged as the leading contributor in terms of the publication volume. miR-21, miR-34a, and miR-195-5p were among the most frequently studied miRNAs, underscoring their relevance to CRC biology and therapy. Keyword and citation analyses identified key thematic areas, such as cell proliferation, epithelial–mesenchymal transition, and chemoresistance, especially to oxaliplatin and 5-fluorouracil. Emerging research frontiers included ferroptosis, ceRNA networks, and exosome-mediated miRNA transport. An analysis of the collaborations indicated strong intra-national collaborations, with room for expanding international research networks. **Conclusions**: This study provides an in-depth bibliometric landscape of the CRC-related miRNA research by highlighting influential studies and journals while identifying gaps and underexplored topics. These insights offer valuable guidance for future translational and clinical research on this topic.

## 1. Introduction

Colorectal cancer (CRC) is one of the most common malignancies worldwide, ranked as the third most frequently diagnosed cancer and the second leading cause of cancer-related death [1]. Despite significant advances in screening and therapeutic strategies, the global burden of CRC continues to grow, and many patients are still diagnosed at late stages when effective treatments are limited and survival outcomes are poor [2]. The multifactorial etiology of CRC includes genetic mutations, epigenetic alterations, chronic inflammation, and environmental factors, but the intricate molecular networks and mechanisms underlying tumor initiation, progression, and metastasis still have not been completely elucidated [3]. In recent years, microRNAs (miRNAs), as small non-coding RNAs that regulate gene expression post-transcriptionally, have received attention due to their pivotal roles in pathophysiology mechanisms and their potential as minimally invasive biomarkers and therapeutic targets [4,5,6]. miRNAs are approximately 18–25 nucleotides in length and develop from endogenous transcripts through a multistep biogenesis pathway involving Drosha and Dicer-mediated processing [7]. Incorporated into the RNA-induced silencing complex (RISC), mature miRNAs bind complementary sequences in target messenger RNAs (mRNAs), leading to translational repression or mRNA degradation [8].

Since their discovery, miRNAs have emerged as pivotal regulators within the intricate framework of gene expression, enhancing our understanding of post-transcriptional control mechanisms. To date, more than 2500 human miRNAs have been identified (miRBase v 22), each capable of modulating multiple target genes and thereby orchestrating complex regulatory networks across a wide range of biological processes [9]. These small non-coding RNA molecules exert their influence not only on mRNAs but also on a broader spectrum of RNA species, including long non-coding RNAs (lncRNAs), pseudogenes, and circular RNAs (circRNAs), forming elaborate competitive endogenous RNA (ceRNA) networks [10].

Through these interactions, miRNAs control gene expression, acting as both repressors and modulators of transcript stability and translation. The regulatory complexity is due to the unique characteristics of miRNAs, as a single miRNA can influence hundreds of transcripts, while a single mRNA may be targeted by multiple miRNAs [11]. Indeed, miRNAs provide a dynamic and responsive control system essential for maintaining cellular homeostasis, but when dysregulated, the same mechanisms contribute to the pathogenesis of various diseases, especially cancer [12,13,14].

In oncology, miRNAs have raised critical attention for their dualistic roles as either oncogenic miRNAs (onco-miRs) or tumor-suppressive miRNAs (TS-miRs), depending on their context-specific expression and functional targets [15]. By modulating key processes such as cell viability, death, and invasion, miRNAs critically influence tumor development and progression [4,16].

Moreover, the ceRNA hypothesis underscores how changes in one component of the network, such as overexpression of a lncRNA sponge or disruption of a circRNA decoy, can have widespread effects, triggering oncogenic activity or reestablishing tumor suppression [17,18].

Beyond their intracellular roles, miRNAs are actively secreted within exosomes or microvesicles, are bound to protein complexes, and are stable in serum, plasma, and other biofluids [19]. This extracellular miRNA fraction has a significant role as a minimally invasive biomarker platform for early cancer detection, disease monitoring, and real-time assessment of treatment efficacy [20]. Several miRNAs are already present in advanced clinical trials, either as diagnostic tools or as synthetic mimics and inhibitors designed for targeted delivery, underscoring the translational potential of the research topic [5,21,22].

Given their central role in gene regulatory networks and disease biology, the miRNA research area has expanded rapidly over the past two decades. To gain an understanding of this research topic, bibliometric analysis can serve as a valuable tool for evaluating trends in the scientific literature, pinpointing influential studies, and mapping research collaborations [23,24,25]. By examining extensive datasets of academic publications, bibliometric analysis can identify key studies, dominant research topics, and emerging areas of interest over time. This approach employs quantitative metrics, such as citation counts and publication frequencies, to assess the impact and relevance of specific works, authors, journals, and institutions. Additionally, bibliometric analysis offers insights into the structure of scientific networks by uncovering collaboration patterns among researchers and institutions across various regions and disciplines. It aids in identifying key contributors and influential groups propelling advancements in a field, as well as the geographic distribution of research activities [26,27].

This bibliometric analysis aims to systematically map the scientific landscape of miRNA research, with a particular focus on its relevance to CRC biology and therapeutic potential. Utilizing bibliometrics, we provide a quantitative perspective of the development of this topic and indicate future investigations focused on the most promising trends in miRNAs in CRC.

## 2. Materials and Methods

### 2.1. Dataset Identification and Screening

**Figure 1:**
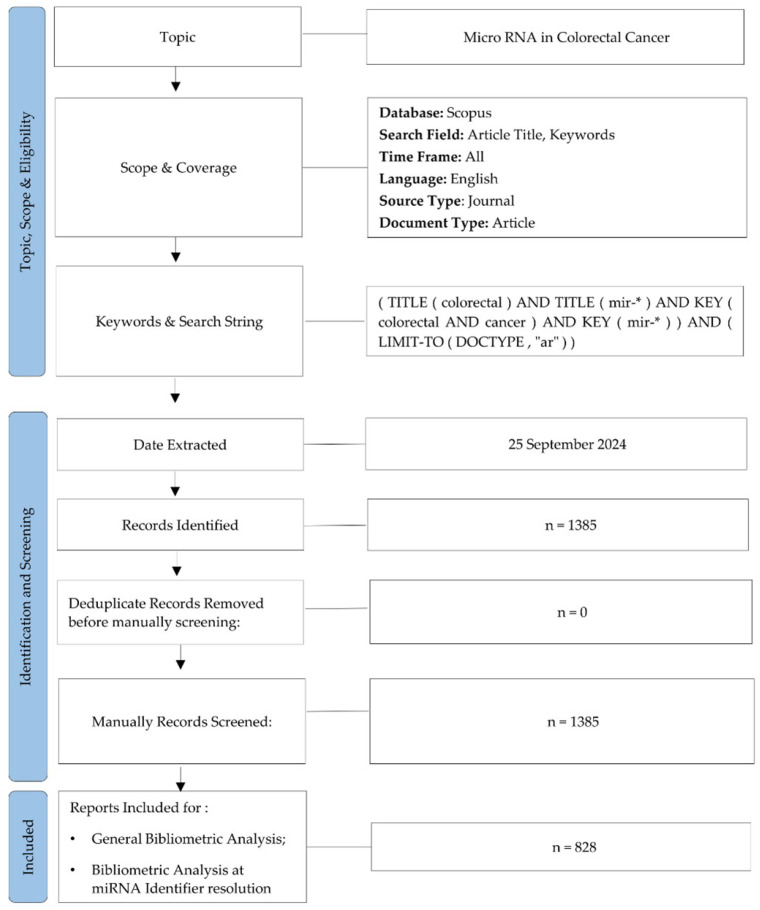
Workflow diagram of this study. Schematic illustration of the methodological steps, including systematic screening of records, data collection, and analysis processes to ensure rigor in the bibliometric evaluation. In the search query, the asterisk (*) is used as a wildcard character in Scopus to retrieve all microRNA identifiers beginning with “mir-”. n: number of publications. N.b. Records were manually screened.

This bibliometric analysis was conducted using data retrieved from the Scopus database, a widely recognized platform frequently utilized in academic and bibliometric research for its multidisciplinary coverage and robust citation metrics [28]. Scopus ensures extensive multidisciplinary coverage and delivers reliable citation metrics, facilitating in-depth analyses of research topic evolution and emerging academic trends [29]. The search strategy was focused on two primary keywords: microRNA and colorectal cancer, including standardized abbreviations to ensure retrieval of the relevant literature. To minimize off-topic results, keywords were restricted to titles and author-provided keywords, excluding abstracts. The final search syntax, optimized for specificity, was formulated as follows: (TITLE (colorectal) AND TITLE (mir-*) AND KEY (colorectal AND cancer) AND KEY (mir-*)) AND (LIMIT-TO (DOCTYPE, “ar”)) (accessed on 25 September 2024) (Figure 1). 

The analysis encompassed all English-language articles published in Scopus and was restricted to original research studies. Review articles and meta-analyses were systematically excluded to focus on primary research. Manual review excluded off-topic studies by screening titles, keywords, and abstracts. Additionally, studies that did not explicitly examine miRNA expression, function, or biomarker potential in CRC were excluded, regardless of their methodologies or outcomes. Both the raw Scopus dataset and the curated subset of relevant records are publicly accessible on GitHub (https://github.com/MichelangeloAloisio/Bibliometric_Analysis_miRNA_CRC/) (accessed on 19 March 2025).

### 2.2. Bibliometric Analysis

The bibliometric analysis was performed using the R software (Version 4.3.3) [30] and its R/bibliometrix package (Version 4.3.5) [26], accessed via its user-friendly Biblioshiny interface. This tool integrated statistical and mathematical methods to analyze bibliometric datasets, enabling a multidisciplinary exploration of the scientific landscape surrounding miRNAs and CRC. Specifically, the Scopus dataset containing only relevant articles was uploaded on R/bibliometrix by using the Surname and Initials of Author Name Format.

A temporal analysis of annual publications and citation trends was performed to assess the evolving impact of the research over time.

Notably, concerning the citation trend, to assess the impact of key publications, both local and global citation analyses were performed. Specifically, local citations (LCSs) refer to the number of times a document was cited by other documents within the study dataset, reflecting its influence within the specific research domain. Global citations (GCSs), retrieved directly from the Scopus database, represented the total citation count across all indexed publications, indicating broader scientific impact. This dual perspective, combining global visibility with local centrality, provides a more comprehensive assessment of the scientific impact, distinguishing between widely recognized milestones and core references that have shaped the trajectory of research.

Moreover, to enhance the resolution of the annual publications at the miRNA ID level, a customized Python script (version 3.10.9) was developed. The script, available at (https://github.com/MichelangeloAloisio/Bibliometric_Analysis_miRNA_CRC/) (accessed on 19 March 2025), systematically extracted miRNA names from (i) the title, (ii) author keywords, and (iii) the abstract of each dataset record. To ensure accuracy, extracted miRNA names were validated against all known miRNA entries using the miRBase database (releases 20, 21, and 22) [31]. This validation process cross-referenced the extracted miRNA identifiers with the miRBase nomenclature to exclude non-standard or outdated terms, ensuring current biological and terminological standards. The tool further quantified the annual occurrence of each miRNA and identified the most frequently studied miRNAs over time. The rankings were determined using two metrics: (1) the cumulative occurrence count across all years and (2) an average calculated from the first publication year. This approach enables an analysis of temporal trends and miRNA-specific relevance to the topic.

A “Country Scientific Production” plot was generated to illustrate the global distribution of the publications. This metric attributed each article to all co-authors’ countries, thereby counting the number of author affiliations per country. The dataset was generated using the R/bibliometrix package, while the plot was created with the rworldmap library (version 1.3.8) [32] to visualize the geographical distribution. The full pipeline is available at https://github.com/MichelangeloAloisio/Bibliometric_Analysis_miRNA_CRC/ (accessed on 19 March 2025). Additionally, the percentage contribution of each country was calculated to highlight their relative involvement in the research topic.

As an alternative analysis related to the country dimension, the analysis of the corresponding author’s country was performed using the default setting of the top 20 countries in the dataset. This method attributes each article to the Single-Country Publications (SCPs), based on the affiliation of the corresponding author, with the frequency representing the total number of articles per country. Additionally, the analysis calculated the Multiple-Country Publication (MCP) index, which quantifies the proportion of articles where at least one co-author has an affiliation in a different country from that of the corresponding author [26,33].

A country-level collaboration network plot was developed to examine global research partnerships between miRNA and CRC. The Walktrap clustering algorithm was employed to detect clusters of countries with similar collaboration patterns, using an automatic layout and association as the normalization method. Key parameters were set to include a maximum of 30 nodes, a repulsion force of 0.1, and a minimum of one edge per node, while excluding isolated nodes.

In the resulting network, nodes correspond to countries, and their sizes reflect publication counts. Edges represent co-authorship relationships, where the thickness indicates the strength of collaboration. Clusters, differentiated by color, group countries sharing similar collaboration patterns.

A temporal analysis of annual publication and citation trends was performed to assess the evolving impact of the research over time. Moreover, to enhance the resolution of the annual publications at the miRNA ID level, a customized Python script (version 3.10.9) was developed. The script, available at (https://github.com/MichelangeloAloisio/Bibliometric_Analysis_miRNA_CRC/) (accessed on 19 March 2025), systematically extracted miRNA names from (i) the title, (ii) author keywords, and (iii) the abstract of each dataset record. To ensure accuracy, extracted miRNA names were validated against all known miRNA entries using the miRBase database (releases 20, 21, and 22) [31]. This validation process cross-referenced the extracted miRNA identifiers with the miRBase nomenclature to exclude non-standard or outdated terms, ensuring current biological and terminological standards. The tool further quantified the annual occurrence of each miRNA and identified the most frequently studied miRNAs over time. The rankings were determined using two metrics: (1) the cumulative occurrence count across all years and (2) an average calculated from the first publication year. This approach enables an analysis of temporal trends and miRNA-specific relevance to CRC.

The 10 Most Relevant Affiliations plot was produced using the affiliation Name Disambiguation option.

The authors’ keywords were analyzed using text analysis methods. A “Word Cloud” visualization was created to display the frequency distribution of these keywords, where the size of each term reflects its prevalence in the dataset. The visualization included the top 100 keywords, excluding terms removed during Scopus dataset extraction and their synonyms. The word occurrence scaling was settled as frequency values. Moreover, a keyword co-occurrence network was generated using VOSviewer (version 1.6.20) [34] to map the conceptual structure of miRNA in CRC research. The analysis included keywords occurring at least five times. The network was constructed using the full counting method and the fractionalization normalization approach to weight co-occurrence relationships. Clusters were identified using the VOS clustering algorithm with a resolution of 0.5 and a minimum cluster size of 10. Node size reflects keyword frequency, and edge thickness indicates the strength of co-occurrence. The layout was optimized with an attraction normalization of 4 and repulsion of 1, ensuring a clear and interpretable visualization of the research landscape.

## 3. Results

### 3.1. Publication and Citation Trends

The bibliometric analysis was conducted on 828 publications retrieved from the Scopus database using a comprehensive query (Figure 1).

**Figure 2:**
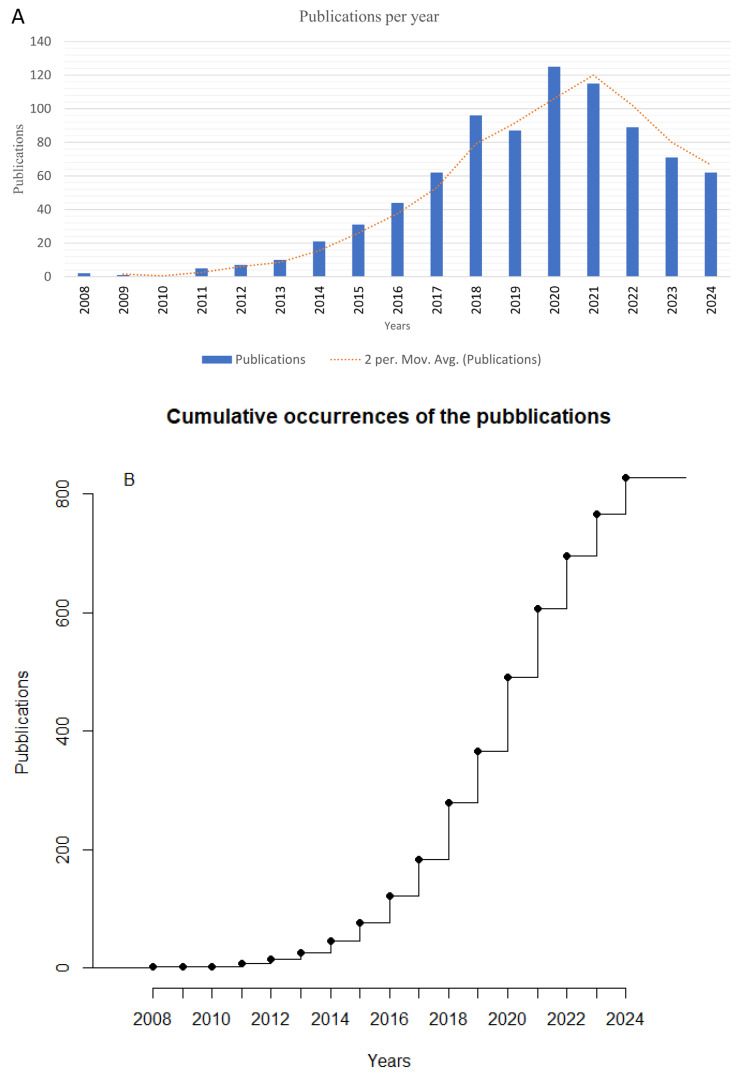
(**A**) Publications per year. The evolution in research publications regarding the connection between CRC and miRNAs can be traced through the rising count of such publications. The orange dashed line represents a 2-year moving average trendline, highlighting the underlying growth trajectory. (**B**) Cumulative number of publications across years. N.b. The number of publications in the year 2024 was incomplete due to the analysis timeline.

Initially, 1385 records were extracted, but after excluding 557 off-topic papers, the final dataset comprised 828 articles focused on miRNA and CRC research (Figure 1). These studies spanned the period from 2008 to 2024, with data accessed up to September 2024. The annual average growth rate of publications, calculated using bibliometric tools, was 23.94% (Table S1), indicating a significant increase in research output over the years. Figure 2A,B and Table 1 collectively summarize the studies in detail, including citation data that demonstrate their impact and significance in scientific research. Together, they also identify three critical phases in miRNA and CRC research, mapping the trajectory of scientific activity across the study period. The first period (2008–2010) was marked by limited activity, with fewer than five publications annually. The next phase (2011–2020) was characterized by a significant increase in research output, rising from 5 publications in 2011 to 125 in 2020. This was followed by a declining phase (2021–2024), during which the annual number of papers per year was declining.

### 3.2. Distribution of Annual Publications Based on miRNA Identifier Level

**Figure 3:**
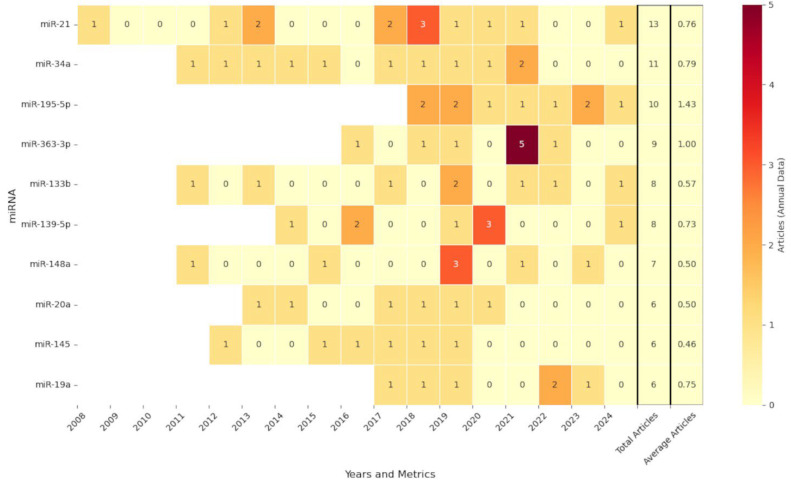
Annual miRNA heatmap. It illustrates the publication trends of the top 10 most frequently referenced miRNAs in the scientific literature related to CRC, arranged in descending order based on the total number of articles across all years. Each row corresponds to a specific miRNA, while the columns denote the years ranging from 2008 to 2024. The values within the heatmap represent the number of scientific articles published in a given year that address the respective miRNA. Additionally, the final two columns provide the total number of articles and the annual mean number of articles. The 2024 articles were considered up to the month of September.

Using a customized Python script (version 3.10.9) [35], 484 miRNA identifiers (IDs) were extracted by title, author keywords, and abstracts from the 828 relevant articles (see Section 2). The IDs were validated against all known entries in the miRBase database [31]. Subsequently, the annual occurrence of each miRNA was counted for the 2008–2024 period. Figure 3 shows the annual miRNAs heatmap ranking the top 10 miRNAs in descending order of total articles. The full table is available in Table S2. miR-21 was the most frequently studied, evaluated in 13 articles between 2008 and 2024.

### 3.3. Most Cited Documents

The top 10 most cited articles on miRNA dysregulation in CRC are listed in Table 2, and the full table is available in the Supplementary Material (Table S3). The highest number of citations (*n* = 1669) was achieved by the 2008 article “MicroRNA-21 (miR-21) post-transcriptionally downregulates tumor suppressor Pdcd4 and stimulates invasion, intravasation and metastasis in colorectal cancer” [36], published in the Oncogene journal, with a 7.22 impact factor (IF) at the time of publication. The most cited articles (total citations = 186–1669) spanned publication years from 2008 to 2020. Among these articles, Asangani IA et al. (2008) [36] achieved 92.72 Total Citations per Year (TC/Y), with 77.5 for Zhao S et al. (2020) [37] and 49.44 for Han P et al. (2017) [38].

Moreover, one article [37] in this list was published in Q1 journals with an impact factor greater than 10 (Journal of Hematology & Oncology), reflecting the substantial scientific influence.

The global citation metrics in Table 2 highlight the broad impact of key studies across the oncology research community. However, to better understand the internal structure and influence, we also performed a local citation analysis, which identifies works most frequently cited within the analyzed dataset.

**Figure 4:**
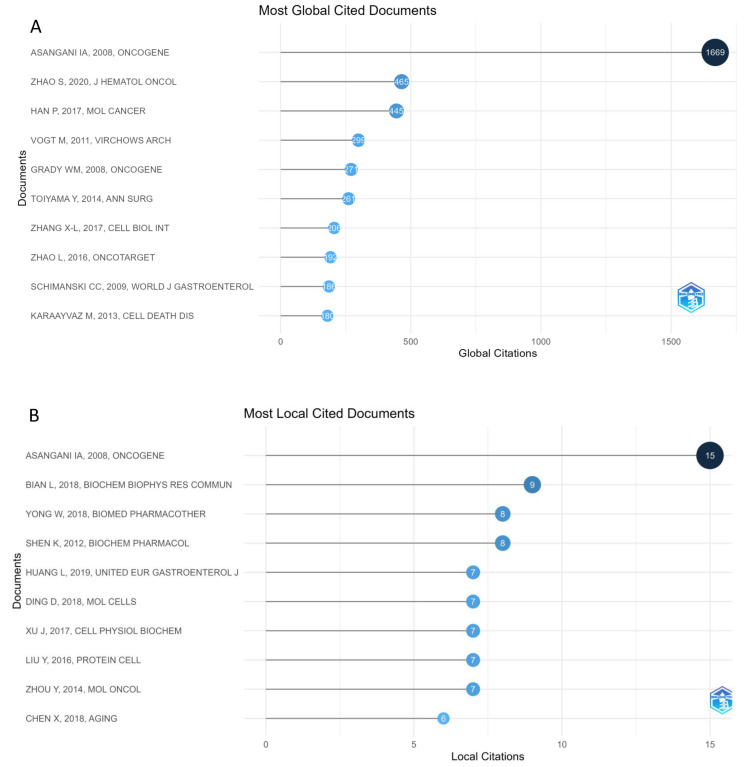
(**A**) List of the most globally cited documents on miRNA research related to colorectal cancer (CRC), ranked by their global citation counts. Each document is labeled with the author(s) names, publication year, and journal [36,37,38,39,40,41,42,43,44,45]. (**B**) List of the most locally cited documents on miRNA research related to CRC, ranked by their local citation counts. Each document is labeled with the author(s) names, publication year, and journal [36,46,47,48,49,50,51,52,53,54].

Figure 4 highlights key works that have significantly influenced the research topic, providing insights into key contributions. Specifically, Figure 4A shows the global citations, i.e., the total citations that an article, which was included in the study dataset, has received from documents indexed on the Scopus database. In other words, the global citations count citations received by a selected article “all over the world”. Figure 4B highlights key studies that have been most frequently cited within the analyzed dataset, reflecting their influence on the specific research topic. In fact, local citations are citations received by a reference article considering only the articles included in the analyzed dataset. 

### 3.4. Geographical Distribution and Collaboration

**Figure 5:**
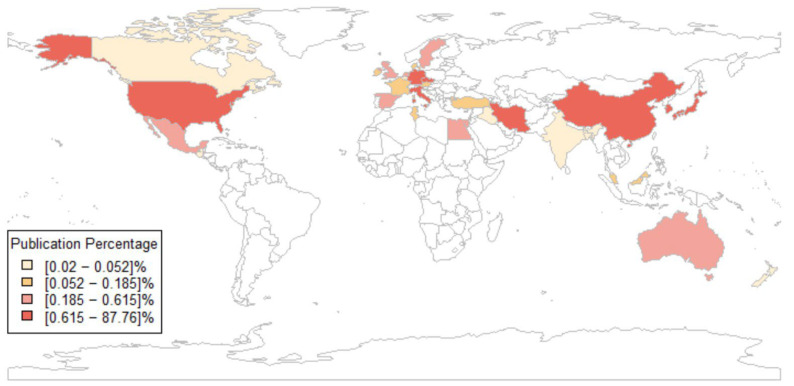
Country Scientific Production plot. The global distribution of publications (in percentage) concerning the relationship between miRNAs and CRC. Countries without contributions in this field are shown in white. The color scale reflects the number of authors’ appearances by affiliation in each country.

Thirty nations have contributed to publications on the relationship between miRNAs and CRC, as measured by the number of author appearances by the country of affiliation. This metric attributes each article to the co-authors’ countries, resulting in a total of 5670 author appearances. China dominates this ranking, with 4976 appearances (87.76%), followed by Iran (118; 2.08%), Japan (114; 2.01%), Italy (75; 1.32%), and the United States (68; 1.2%). The remaining countries contribute less than 1% each (Figure 5). Notably, China’s overwhelming presence suggests a combined research effort in this area. The full list of contributing countries is available in Table S4.

**Figure 6:**
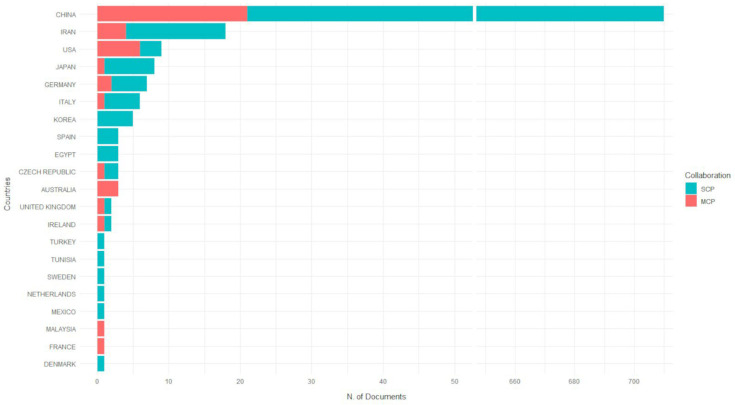
Corresponding authors’ countries. This chart illustrates the distribution of SCP articles attributed to a single nation via the corresponding author’s affiliation (blue bars) and MCP collaborative works involving international co-authors (red segments). The broken *x*-axis is used to graphically represent the large disparity in publication counts between China and other countries.

To analyze leadership in research output, Figure 6 considers the corresponding authors’ countries, attributing each article to a single country. Here, China shows its dominance, with 710 corresponding authorships (85.7% of 828 total articles), followed by Iran (18; 2.2%), the United States (9; 1.1%), Japan (8; 1.0%), and Germany (7; 0.8%). Notably, China’s corresponding authorships (710) align closely with its SCP total (689), and only 3% involve MCPs (21 articles) (Table S5); the corresponding author’s country analysis associated articles to a single country on the basis of the affiliation of the corresponding author (i.e., SCP). In this case, the frequency per country corresponded to the total number of articles. In addition, this analysis also calculated the proportion of articles in which there was at least one author with an affiliation in a country other than that of the corresponding author: this index is called MCP. It is worth noting that 41 articles (5% of the total) were excluded from the corresponding author analysis due to missing affiliation data. In contrast, countries like the United States (MCP: 8; 66.7%), Australia (MCP: 3; 100%), and France (MCP: 1; 100%) exhibit high rates of international collaboration, as indicated by their MCP values. These values reflect a significant proportion of articles involving co-authors affiliated with countries different from the corresponding author’s country. However, these percentages reflect small absolute numbers (e.g., Australia: three articles in total, all MCP; France: one article, MCP; Malaysia: one article, MCP), highlighting that, while collaboration rates are high, the overall contribution of these nations remains limited.

**Figure 7:**
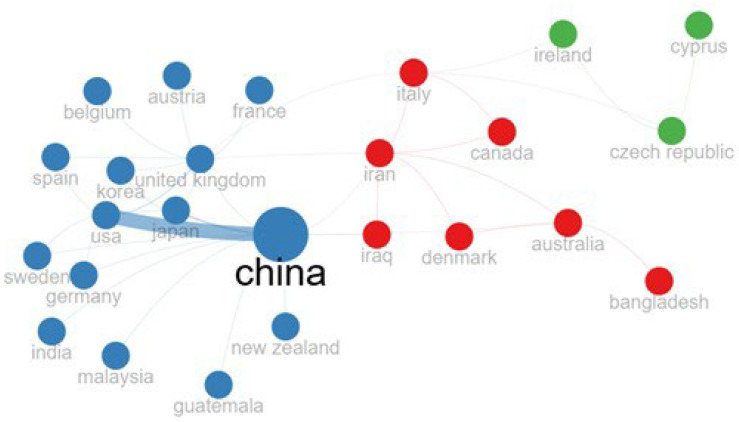
Collaboration network by country. Map of international collaboration networks in CRC and miRNAs, revealing three distinct clusters (color-coded) with shared patterns.

Figure 7 illustrates the global collaborative network in scientific research on miRNAs and CRC, revealing three distinct clusters (color-coded) that reflect groups of countries with shared patterns of collaborative activity.

China emerges as the primary hub in this network, characterized by an exceptionally high frequency of international collaborations. It exhibits robust collaborative links, principally with the United States but also with Japan, the United Kingdom, Germany, Sweden, India, Malaysia, Guatemala, and New Zealand. This connection highlights its pivotal role in advancing global research efforts on this topic. The green cluster (encompassing Ireland, the Czech Republic, and Cyprus) appears more isolated, indicating limited integration into the broader collaborative network.

### 3.5. Most Productive Journals

**Figure 8:**
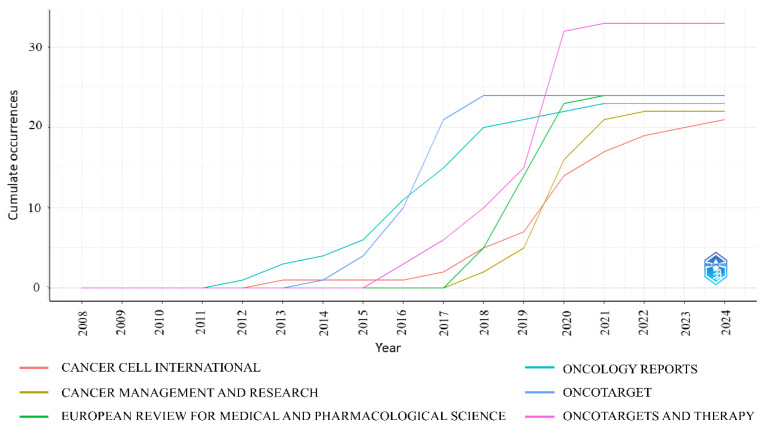
Sources of production journals over time. This chart illustrates the cumulative growth in article publications by the six most popular journals from 2008 to the end of 2024. Each line represents the journal’s contribution to the topic, based on the total publication volume.

The analysis identified six journals that collectively published the majority of articles on miRNAs and CRC (Figure 8), each contributing ≥ 20 articles. The most prolific journals include Oncotargets and Therapy (33 articles in 2024), European Review for Medical and Pharmacological Sciences (24 articles), Oncotarget (24 articles), Oncology Reports (23 articles), Cancer Management and Research (22 articles), and Cancer Cell International (21 articles). A sharp increase in publications is observed after 2011, reflecting intensified research focus on miRNA–CRC interactions. The full list of 50 journals analyzed, including annual publication trends, is provided in Table S6.

### 3.6. Most Productive Institutions

**Figure 9:**
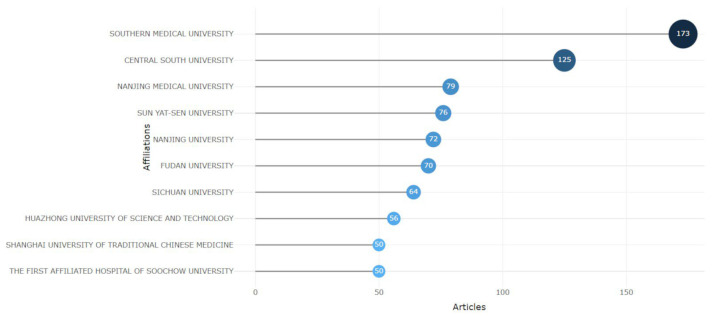
Most Relevant Affiliations. The 10 most prolific organizations on miRNAs and CRC.

Figure 9 illustrates the top 10 prolific organizations with 50 or more documents published. In the top ranking are “Southern Medical University” with 173 publications, “Central South University” with 125 publications, and “Nanjing Medical University” with 79 publications. Interestingly, the top ten productive institutions that have published studies related to miRNA in CRC research are in China.

### 3.7. Word Cloud Frequencies

**Figure 10:**
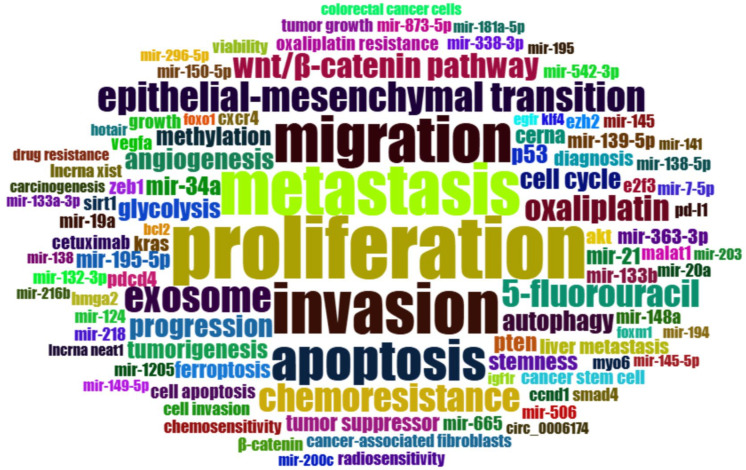
Word cloud plot. The 100 most frequent keywords related to miRNAs and CRC. The size of each word reflects its frequency and relevance within the dataset.

The keyword analysis was focused on identifying the most frequently used keywords by the author in the extracted dataset, excluding terms originally part of the Scopus research query. The list of synonymous terms is detailed in Table S7, while the excluded terms are listed in Table S8. The findings are summarized in the word cloud plot (Figure 10), which visually highlights the most common author keywords associated with CRC and miRNAs. In this visualization, the size of each word reflects its frequency and relevance in the dataset: larger terms represent widely studied topics, while smaller terms indicate underexplored areas that could benefit from further research.

The word cloud frequency revealed a strong focus on cellular mechanisms driving CRC progression, with proliferation (117 occurrences) and metastasis (79) being the most frequently studied processes. These terms highlight intense research into tumor growth and dissemination, often linked to invasion (77), migration (64), and the epithelial–mesenchymal transition (EMT) (27), which collectively underpin metastatic potential. Apoptosis (51) and angiogenesis (13) further underscored investigations into programmed cell death and tumor vascularization. The Wnt/β-catenin pathway (19) and cell cycle (12) dominated discussions of oncogenic signaling, while autophagy (12) and glycolysis (11) reflected metabolic reprogramming in CRC. Emerging areas included ferroptosis (8), an iron-dependent cell death mechanism, and Cerna (8), a competing endogenous RNA regulatory network. Therapeutic and diagnostic targets such as PD-L1 (5), radiosensitivity (5), and KRAS (7) mutations pointed to advances in immunotherapy and precision medicine. Genetic and epigenetic drivers like TP53 (10), methylation (10), and PTEN (10) were frequently linked to tumorigenesis (10). While exosomes (27) were prominent due to their roles in miRNA transport, terms like MALAT1 (6) and lncRNA XIST (5) signaled growing interest in non-coding RNAs. Despite this, ferroptosis and Cerna remained underexplored, suggesting opportunities for future research. The complete frequency distribution of all 100 keywords is available in Table S9.

**Figure 11:**
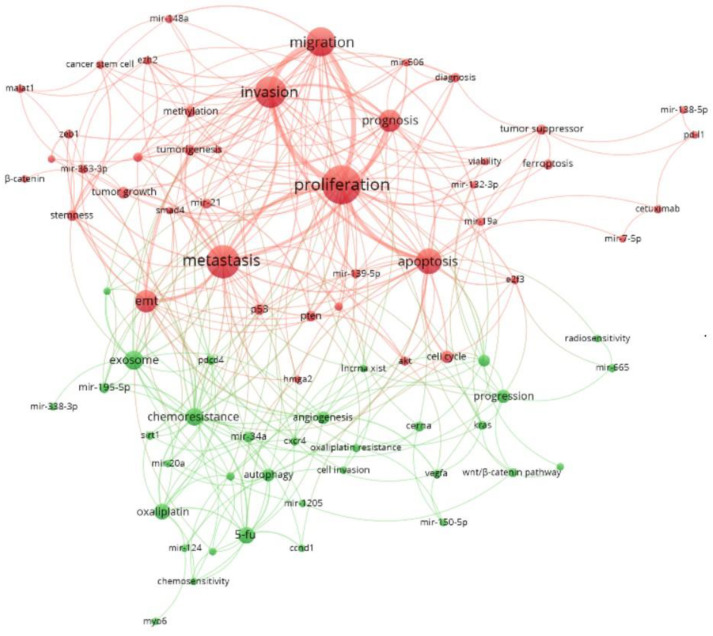
VOSviewer-generated network map of co-occurring author keywords related to miRNA and CRC. Node size indicates keyword frequency, colors represent thematic clusters, and link thickness reflects the strength of co-occurrence, illustrating the conceptual structure and thematic interrelations within the field.

A network visualization of author keyword co-occurrence related to miRNA and CRC was generated using VOSviewer. Author keywords were extracted from 828 publications and standardized using a custom thesaurus file to ensure term consistency (Table S10). Non-informative and general terms were removed to enhance thematic clarity, with the complete list of excluded keywords provided in Table S8. The distinct colors in the network (Figure 11) represent different thematic clusters: the red cluster encompasses core biological processes such as “proliferation,” “metastasis,” “invasion,” and “apoptosis,” highlighting their centrality and high prevalence in the current literature. In contrast, the green cluster includes emerging and specialized themes, such as “chemoresistance,” “angiogenesis,” and “autophagy”, indicating growing research interest in tumor microenvironment regulation and therapy-related mechanisms.

Node size reflects the frequency of keyword occurrence, indicating the relative prominence of each topic, while the links between nodes represent co-occurrence patterns. Thicker lines denote stronger associations, reflecting closely related research themes. Within the red cluster, the strong interconnections among “metastasis,” “migration,” and “invasion” emphasize a concentrated focus on tumor progression and metastatic dissemination. Similarly, in the green cluster, the dense linkages between “chemoresistance,” “oxaliplatin,” and “autophagy” suggest a significant interplay between drug resistance pathways and key cellular processes in cancer biology. Overall, the network reveals a well-defined research landscape, characterized by a central core of molecular oncology topics (red cluster) and peripheral themes related to therapeutic challenges and adaptive responses (green cluster). The strength of connections across clusters reflects the high degree of integration among key concepts, underscoring the interconnected nature of current research in miRNA and cancer.

This bibliometric mapping not only identifies the dominant research themes but also captures the evolving trajectory of the field. The integration of specialized and peripherally positioned concepts into central biological processes highlights a research domain that is both firmly grounded in fundamental oncology and dynamically advancing toward novel mechanistic insights and translational applications.

## 4. Discussion

This study presents the first bibliometric analysis examining the impact of miRNAs in CRC. A distinctive feature of this research is the initial article selection conducted through Scopus [28]. The analysis covers the period from 2008 to September 2024 and was carried out using the R/bibliometrix package and the biblioshiny web interface [26].

The bibliometric analysis of miRNA research in colorectal cancer (CRC) clearly revealed three distinct phases in the publication trends in this research topic over the years. During the first period (2008–2010), scholarly experience of this topic was limited, and fewer than five articles were published annually. However, those studies achieved high visibility, reaching a mean of approximately 54 citations per paper in 2008. This unbalanced impact probably reflects pioneering efforts to establish the functional relevance of miRNAs in CRC biology. In fact, the absence of publications in 2010 may suggest that technical limitations and a still-developing conceptual framework limited the discovery of new findings and their dissemination within the scientific community. In this phase, three pioneering studies established both mechanistic and translational frameworks for miRNA biology in CRC, exerting influence across multiple oncology disciplines. Asangani et al. showed that miR-21 promotes CRC invasion and metastasis by targeting Pdcd4. The discovery of RISC-mediated regulation of tumor invasion rapidly influenced studies on breast, lung, and glioma cancers, explaining its exceptionally high citation count (1669 citations to date; Table 2) [36]. In the same year, Grady et al. identified epigenetic silencing of miR-342 in CRC, highlighting DNA methylation’s role in miRNA regulation, attracting interest across cancer epigenetics studies (271 citations) [40]. Schimanski et al. correlated miR-196a expression with tumor stage and lymph node involvement, establishing tissue and circulating miRNA profiling as a prognostic tool in CRC; its clinical-marker focus led to cross-disciplinary citations in gastroenterology, pathology, and biomarker development (186 citations) [44]. Collectively, these works introduced key regulatory paradigms, validated miRNAs as biomarkers, and sparked extensive interdisciplinary follow-up studies. This critical transitional phase was followed by a period of rapid growth, highlighting a substantial increase from 11 publications in 2011 to 125 in 2020. This trend was also supported by the total citation rate that grew over time, peaking at 664 in 2020 and underscoring both the rise in research productivity and its uptake by the broader community. At the same time, the gradual decline in average citations per article (from 10.4 in 2011 to 5.3 in 2020) suggests that the new papers had a short amount of time to gain more citations, compared to earlier publications that benefited from a longer citation window, yielding higher averages.

The trendline illustrates this sustained growth, characterized by methodological improvements, broader collaboration among researchers, and enhanced clinical interest in the diagnostic and therapeutic potential of miRNAs. During the Peak Activity Phase (2021–2024), research output reached a sustained high level, and annual publications consistently exceeded 62 studies. A marked decline in average citations per publication was highlighted in this period due to the natural delay in citation accumulation for recent articles and the steady and very high competition in this topic.

The top 10 most frequently referenced miRNAs underscored their impact on the significant development of the research topic, suggesting their potential role in CRC pathophysiology and therapy. miR-21 was the most frequently studied miRNA in CRC (13 articles spanning 2008 to 2024) that was characterized as a tumor suppressor early on, as its expression was found to be significantly downregulated in CRC cell lines and tissue specimens. Increasing levels of miR-21 inhibited cancer cell growth, migration, invasion, and angiogenesis through VEGF expression [55]. miR-21 facilitates CRC progression by repressing PTEN post-transcriptionally, promoting cell growth and invasion; anti-miR-21 strategies have been shown to restore PTEN levels and mitigate malignancy [56]. This deep involvement in CRC pathogenesis has positioned miR-21 as a key target of interest for both fundamental mechanistic studies and translational applications, including biomarker development and therapeutic strategies. Beyond miR-21, miR-34a and miR-195-5p have shown similarly high levels of research attention, each with up to 10 articles or more published within the analyzed timeframe. miR-34a, a well-established downstream effector of the tumor suppressor p53, contributes to the regulation of apoptosis and cell cycle arrest in CRC models [57,58]; miR-195-5p upregulation inhibits CRC proliferation via the downregulation of CDK2/CDK8 and the anti-apoptotic protein BCL2L2 [59] or by repressing FGF2 and Wnt/β-catenin signaling to inhibit tumor growth [60,61]. In addition, considerable evidence showed novel regulatory mechanisms controlled by miR-195-5p in the modulation of adhesive junctions and keratins implicated in CRC development and progression, affecting cell proliferation, viability, invasion, and in vivo growth. Specifically, the tumor-suppressive role of miR-195-5p in CRC has been demonstrated, revealing it as a critical regulator of multiple cellular processes and identifying γ-catenin (JUP) [62], keratin 23 (KRT23) [63], and pinin (PNN) [64] as its major downstream targets. These proteins are functionally associated with desmosomes and are involved in cell adhesion, cytoskeletal dynamics, and oncogenic signaling. They play pivotal roles in maintaining epithelial integrity and promoting cancer cell proliferation and dissemination when dysregulated. By suppressing these targets, miR-195-5p disrupts pro-tumorigenic mechanisms, reducing CRC progression. In addition, the in vivo delivery of miR-195-5p mimics in preclinical models resulted in a significant reduction in tumor growth, pointing to its promise as a novel potential RNA-based therapeutic agent [65].

miR-27a-3p expression was found to be affected by lncRNA RMST, which acted as a competitive endogenous RNA. Hence, RMST suppresses colorectal cancer progression by regulating the miR-27a-3p/RXRα axis and blocking Wnt signaling [66]. Zhang X showed that miR-133b reduced cancer cell stemness and decreased chemoresistance to treatments like 5-fluorouracil by inhibiting DOT1L [67]. The increase in miR-19a suppressed the oncogene KRAS, leading to a reduced formation of new blood vessels, limiting tumor growth and spread in colorectal cancer [68]. Another study on miR-19a underscored its role in promoting CRC cell proliferation and migration and facilitating tumor growth in vivo by targeting TIA1 [69].

As publication volume has grown over time, citation activity has also increased. The most cited paper, “MicroRNA-21 (miR-21) post-transcriptionally downregulates tumor suppressor Pdcd4 and stimulates invasion, intravasation and metastasis in colorectal cancer,” [36] published in Oncogene in 2008, has accumulated 1669 citations, underscoring the foundational role of miR-21 in CRC pathogenesis. This article also achieved the highest Total Citations per Year (TC/Y), at 92.72, highlighting its influence.

Other highly impactful studies include Zhao et al. (2020) [37] with 77.5 TC/Y and Han et al. (2017) [38] with 49.44 TC/Y. Notably, one article among the top ten [37] was published in journals with impact factors exceeding 10 (Journal of Hematology & Oncology), reflecting both scientific rigor and wide visibility. These citation metrics underscore the key role of specific miRNAs, particularly miR-21 and miR-34a, in advancing molecular understanding and suggesting future investigations in CRC biology.

The geographic distribution of research output on miRNA dysregulation in CRC reveals a concentration of scientific activity within a single country. Chinese scholars are the most prolific contributors, with 710 of the 828 analyzed articles (85.7%) based on corresponding authors’ affiliations. This dominant presence is further underscored by its high rate of Single-Country Publications (SCPs), accounting for 689 of the 710 Chinese articles. The Multiple-Country Publications (MCPs), standing at just 3% (21 articles), indicate an intra-national collaboration trend penalizing international research productivity. Although China remains the leading contributor to CRC–miRNA research, Iran, the United States, and Japan have also played important roles in advancing this research area. Iranian scholars have focused on the identification of miRNAs involved in colorectal cancer progression, metastasis, and treatment response by modulating signaling pathways and acting as diagnostic, prognostic, and intracellular communication mediators. The United States distinguishes itself through high levels of international collaboration and in-depth mechanistic studies on miRNA roles in chemoresistance and metastasis. Japan has made clinically impactful contributions, particularly in the early exploration of circulating miRNAs. These countries, although differing in output volume, contribute significantly to the depth and progress in CRC–miRNA research worldwide. Furthermore, countries such as the United States, Australia, and France exhibit a markedly different collaborative profile. While their total publication counts are comparatively lower, these nations demonstrate a high frequency of international co-authorship. For example, 66.7% of U.S.-affiliated publications involve international collaboration, while both Australia and France show 100% MCPs, although with small sample sizes. These trends suggest that international partnerships, although limited in number, may have a substantial impact on the global scientific dialogue on this research topic.

Despite the relatively low proportion of MCPs in Chinese publications based on the corresponding author’s country, the global collaborative network analysis reveals that China is also actively involved in research projects with international partners, including the United States, Japan, the United Kingdom, Germany, and India. This connectivity underscores China’s dual role as both a prolific and a globally integrated contributor to CRC-related miRNA research.

Institutional productivity further reinforces the dominant role of Chinese research institutions in this domain. Organizations such as Southern Medical University, Central South University, and Nanjing Medical University ranked highest in volume of publication (45% of total), suggesting concentrated national investment and coordinated investigations focused on miRNA and CRC.

The sustained increase in publications suggests a growing impact of the regulatory role of miRNAs in CRC pathogenesis, diagnosis, and therapy. The publication output seen in the top six journals underscores a consolidation of scholarly interest, with journals like Oncotargets and Therapy, European Review for Medical and Pharmacological Sciences, and Oncotarget emerging as primary outlets for CRC-related miRNA studies. Their consistent upward trajectories underscore their role as the principal forums for both mechanistic and translational findings; prospective authors may therefore target these journals to maximize visibility within this specialized community.

Interestingly, independent research groups investigating the same miRNA may report different aspects of its activity since a single miRNA can bind to numerous mRNA transcripts and can thereby modulate multiple distinct signaling cascades and biological processes [70]. Consequently, even when independent researchers investigate the same miRNA, they often report divergent target repertoires/profiles and pathway involvements, reflecting differences in experimental systems, tissue contexts, bioinformatic prediction tools, and validation strategies. For instance, it has been reported that inhibiting miR-363-3p or overexpressing EZH2 could partially reverse the anti-tumor effects of MALAT1 depletion, underscoring the functional axis of MALAT1/miR-363-3p/EZH2 in CRC progression [71]. The same miRNA has also been studied by other researchers, who revealed a CCDC144NL-AS1/miR-363-3p/GALNT7 axis as a driver of CRC cell proliferation and suggested that targeting this lncRNA-mediated circuit may offer a therapeutic strategy in colorectal cancer [72]. The critical role of miR-363-3p has also been reported as an inhibitor of EMT and metastatic progression in CRC, acting through direct targeting of SOX4, which highlights its potential as both a prognostic biomarker and a therapeutic candidate [73].

The keyword analysis identified a deep emphasis on the cellular and molecular mechanisms involved in CRC progression, with “proliferation” and “metastasis” emerging as the most prominent topics. These focal points reflect intensive investigations into tumor growth dynamics and dissemination, further evidenced by high frequencies for “invasion”, “migration”, and “epithelial-mesenchymal transition (EMT)”. Together, these terms illustrate a sustained effort to elucidate how miRNAs regulate key processes that enable cancer cells to detach, migrate, and establish secondary lesions. Concurrently, studies on “apoptosis” and “angiogenesis” reveal ongoing interest in the dual roles of miRNAs in promoting programmed cell death and modulating tumor vascularization, both of which are essential for CRC progression and response to therapy. For instance, miR-1 was early characterized as a tumor-suppressive factor in multiple malignancies, demonstrating its negative role on cell proliferation and migration through the targeting of key genes such as LASP1 and Smad3 [74,75]. In addition, miR-1 expression was found to be significantly reduced in colorectal cancer specimens across stages T2, T3, and T4 compared to adjacent non-tumorous mucosa, suggesting a consistent downregulation during tumor progression [76]. miR-21 overexpression in CRC cell lines significantly enhanced proliferation, colony formation, migration, and invasion targeting PTEN [56]. Moreover, in vivo xenograft models corroborated these findings, with miR-21 overexpression enhancing tumor growth and PTEN restoration attenuating this effect. miR-181a directly targets and downregulates SRCIN1 (SRC kinase signaling inhibitor 1), which normally acts to suppress SRC kinase activity. Loss of SRCIN1 leads to SRC pathway activation, which, in turn, increases VEGF (vascular endothelial growth factor) expression. Functional experiments confirmed that miR-181a enhances angiogenesis both in vitro and in vivo, while reintroducing SRCIN1 or blocking VEGF signaling (e.g., with bevacizumab) reversed these effects [77]. Tsai and co-workers revealed that miR-148a directly inhibits ROCK1 and c-Met, leading to the downregulation of HIF-1α, a key factor in hypoxia-induced cancer progression. This suppression results in decreased VEGF secretion and reduced levels of Mcl-1, promoting apoptosis and inhibiting angiogenesis. Importantly, when combined with bevacizumab (an anti-VEGF therapy), miR-148a significantly enhanced the anti-tumor and anti-angiogenic effects both in vitro and in mouse models [78].

The high frequency of “oxaliplatin”, “5-fluouracil”, and “chemoresistance” reflects a substantial research focus on miRNA-mediated modulation of treatment response. This prominence indicates a growing interest in understanding how specific miRNAs influence cellular sensitivity to oxaliplatin or 5-fluouracil, cornerstones of CRC chemotherapy. For instance, miR-135b, miR-203, and miR-29b have been implicated in enhancing or attenuating oxaliplatin efficacy by targeting pathways involved in DNA damage repair and apoptosis. Elevated miR-135b levels provided resistance against oxaliplatin, and its silencing led to a restoration of cell sensitivity by upregulating the tumor suppressor FOXO1, which, in turn, increased the expression of the pro-apoptotic proteins Bim and Noxa [79]. Han et al. identified miR-203 as a driver of oxaliplatin resistance, finding that its level was markedly upregulated in oxaliplatin-resistant CRC cell lines, while its knockdown restored drug sensitivity. In fact, by downregulating ATM kinase, a pivotal mediator of the DNA damage response, miR-203 impaired the activation of key DNA repair markers (e.g., γH2AX), thereby allowing resistant cells to evade oxaliplatin-induced apoptosis [53]. On the contrary, miR-29b is downregulated in oxaliplatin-resistant colorectal cancer cells, where SIRT1 levels are correspondingly elevated. Restoring miR-29b directly suppresses SIRT1, leading to increased p53 acetylation and the activation of pro-apoptotic pathways, thereby re-sensitizing cells to oxaliplatin both in vitro and in vivo [80]. Recently, Zhang and colleagues demonstrated that the overexpression of miR-519d-3p significantly reduced CRC cell proliferation, enhanced apoptosis, and increased sensitivity to 5-Fu, suppressing PFKFB3, a key glycolytic enzyme involved in cancer cell metabolism and chemoresistance [81]. By inhibiting PFKFB3, miR-519d-3p disrupts glycolysis, reduces energy supply to cancer cells, and enhances 5-fluouracil-induced cytotoxicity. In vivo mouse models further confirmed that miR-519d-3p overexpression reduces tumor growth and improves 5-fluouracil efficacy [81]. Another study has shown the role of miR-338-3p in promoting 5-fluorouracil resistance in p53-mutant colon cancer cells via mTOR, a key regulator of autophagy. Inhibiting miR-338-3p restores mTOR activity, decreases autophagy, and resensitizes cells to 5-fluouracil, underlying its role in the mTOR-autophagy pathway as a potential therapeutic target [82]. Another work demonstrated that the increase in miR-195-5p in chemoresistant CRC cells led to decreased GDPD5 expression, resulting in reduced cell migration and invasion and enhanced apoptosis, as well as markedly improved sensitivity to chemotherapeutic agents such as 5-fluorouracil [83].

The frequent co-occurrence of these keywords suggests that researchers are actively mapping miRNA signatures predictive of chemoresistance and exploring miRNA-based strategies to resensitize tumors. Future works should therefore not only catalog these miRNA–drug relationships more comprehensively but also prioritize functional validation in preclinical models, with a focus on integrating circulating miRNA biomarkers into clinical decision-making for the use of personalized oxaliplatin therapy.

In addition, the keyword occurrence trend also highlights pivotal signaling and metabolic pathways, such as the “Wnt/β-catenin pathway”, “cell cycle” regulation, “autophagy”, and “glycolysis”, all of which reflect miRNA involvement in orchestrating oncogenic signal transduction and metabolic reprogramming.

Less frequent but increasingly notable terms include “ferroptosis” and “ceRNA” networks, suggesting nascent interest in iron-dependent cell death mechanisms and competing endogenous RNA interactions, respectively. Similarly, emerging therapeutic and diagnostic keywords, such as “PD-L1”, “radiosensitivity”, and “KRAS mutations”, point to integrated efforts in immunotherapy and precision medicine. The appearance of “TP53”, “methylation”, and “PTEN” underscores the genetic and epigenetic dimensions of CRC research, while “exosomes” and related terms highlight the recognized role of extracellular vesicles in miRNA transport and intercellular communication. Finally, the modest frequencies of “MALAT1” and “lncRNA XIST” signal growing, but still limited, exploration of long non-coding RNAs in conjunction with miRNAs.

Collectively, these patterns reveal a research topic that is both consolidated around classical oncogenic hallmarks and diversifying into innovative areas. The heavy emphasis on proliferation, metastasis, and EMT reflects established research priorities, whereas the lower frequency of ferroptosis and ceRNA-related studies identifies distinct research opportunities that still require more in-depth investigation.

The conceptual structure of CRC–miRNA research was also analyzed by a co-occurrence network of author keywords that revealed two major thematic clusters. The red cluster, dominated by terms such as proliferation, metastasis, migration, invasion, and apoptosis, reflects a strong research focus on tumor progression and core oncogenic processes. In contrast, the green cluster is characterized by keywords linked to chemoresistance, oxaliplatin, autophagy, and exosomes, which highlights an expanding interest in therapy response mechanisms, drug resistance, and intercellular communication. Together, these interconnected clusters illustrate how CRC–miRNA research remains grounded in key molecular oncology while increasingly advancing toward novel mechanistic insights and clinical applications.

This study has several limitations. First, depending exclusively on a single source, i.e., Scopus, for literature retrieval may limit the breadth, precision, and representativeness of the data. While Scopus is extensive and up-to-date, it may miss relevant studies indexed only in databases like Web of Science (WoS) or PubMed [84]. Future research could improve bibliometric analyses by integrating multiple sources, although this may introduce issues such as duplicate entries and inconsistent formats [85]. For instance, many journals appear in both Scopus and WoS, leading to overlap when using the same search terms [85]. Nonetheless, combining databases can help balance the limitations of any one source. Second, limiting the review to English-language articles may exclude important non-English studies, introducing biases such as the misclassification of document types. Lastly, advances in artificial intelligence (AI) are expected to enhance tools for comparative studies, validation, and sensitivity analysis and may support the adoption of AI-based reporting standards like PRISMA-AI into systematic reviews [86]. Despite these limitations, the clusters identified in this study are consistent with the current understanding of miRNA–CRC research.

## 5. Conclusions

In summary, this bibliometric analysis demonstrates that miRNA research in CRC has progressed from isolated high-impact discoveries to a mature and diversified domain. Through the systematic mapping of the publication trends, keyword co-occurrences, and collaborative networks, we highlighted the advances in the investigations of miRNA functions. A concentrated group of authors and journals has catalyzed mechanistic insights, while keyword trends reveal both well-established priorities and underexploited opportunities. The sustained rise in CRC–miRNA publications reflects growing recognition of these small non-coding RNAs as pivotal regulators of tumor biology and chemoresistance. Notably, studies on miR-21, miR-34a, and miR-195-5p have anchored our understanding of apoptosis, epithelial–mesenchymal transition, and metastasis, while emerging works underscore the roles of specific miRNAs in modulating the response to oxaliplatin and 5-fluorouracil via DNA repair, autophagy, metabolic reprogramming, and apoptotic pathways. Future efforts should leverage these findings to i) perform targeted meta-analyses on core miRNAs; ii) expand investigation into emerging areas such as ferroptosis, ceRNA networks, and exosome biology; iii) enhance functional validation of miRNA–drug interactions, particularly in oxaliplatin and 5-fluorouracil resistance; and iv) promote international and multidisciplinary collaborations to accelerate the clinical translation of miRNA-based diagnostics and therapeutics in colorectal cancer. Overall, this analysis underscores the multifaceted potential of miRNAs in CRC and emphasizes the necessity of interdisciplinary efforts to advance their clinical translation.

## Figures and Tables

**Table 1:** Descriptive statistics of articles and citations related to CRC and miRNAs by year (*n* = 828).

Year	NP	Percentage (%)	TC	TC/NP
2008	2	0.2	108	53.89
2009	1	0.1	11	10.94
2010	0	0.0	0	N.D.
2011	5	0.6	52	10.4
2012	7	0.8	42	5.93
2013	10	1.2	72	7.18
2014	21	2.5	124	5.92
2015	31	3.7	157	5.06
2016	44	5.3	184	4.19
2017	62	7.5	401	6.47
2018	96	11.6	552	5.75
2019	87	10.5	545	6.27
2020	125	15.1	664	5.31
2021	115	13.9	409	3.56
2022	89	10.7	224	2.52
2023	71	8.6	117	1.65
2024 **^^^**	62	7.5	20	0.32

NP: Total number of publications in that year; TC: Total citations of documents published in that year; TC/NP: Ratio of total citations to total number of publications in that year; Percentage (%): Proportion of publications generated in that year relative to total publications (*n* = 828); ^^^: Accessed 25 September 2024; N.D.: Not determined.

**Table 2:** Top 10 most globally cited articles on miRNAs and CRC.

SCR	First Author and Year	Journal IF	TC	TC/Y	miRNA ID	Clinical Relevance
1	Asangani IA, 2008 [36]	Oncogene (7.22, Q1)	1669	92.72	miR-21	Therapeutic target
2	Zhao S, 2020 [37]	J Hematol Oncol (17.39, Q1)	465	77.5	miR-934	Prognostic biomarker
3	Han P, 2017 [38]	Mol Cancer (7.78, Q1)	445	49.44	miR-181a-5p	Prognostic biomarker and therapeutic target
4	Vogt M, 2011 [39]	Virchows Arch (2.49, Q1)	299	19.93	miR-34a, b, c	Diagnostic biomarker
5	Grady WM, 2008 [40]	Oncogene (7.22, Q1)	271	15.06	miR-342	Therapeutic target
6	Toiyama Y, 2014 [41]	Ann Surg (8.33, Q1)	261	21.75	miR-200c	Prognostic biomarker
7	Zhang X, 2017 [42]	Cell Biol Int (1.94 2017, Q1)	206	22.89	miR-138-5p	Therapeutic target
8	Zhao L, 2016 [43]	Oncotarget (5.17, Q2)	192	19.2	miR-138-5p	Therapeutic target
9	Schimanski CC, 2009 [44]	World J Gastroenterol (2.09, Q1)	186	10.94	miR-196a	Prognostic biomarker and therapeutic target
10	Karaayvaz M, 2013 [45]	Cell Death Dis (5.18, Q2)	180	13.85	miR-129	Therapeutic target

Note. SCR = Standard Competition Ranking; Journal IF: refers to the IF at time of publication, sourced from Journal Citation Reports (https://jcr.clarivate.com/jcr/home accessed on 10 April 2025); TC = Total citations; TC/Y = Average citations per year.

## Data Availability

The data and pipeline used in this study are available on GitHub (https://github.com/MichelangeloAloisio/Bibliometric_Analysis_miRNA_CRC/) (accessed on 19 March 2025).

## Supplementary Materials

The following supporting information can be downloaded at: https://www.mdpi.com/article/10.3390/pharmaceutics17081084/s1, Table S1: Main information bibliometrix; Table S2: miRNA name per year; Table S3: Most globally cited documents bibliometrix; Table S4: Country production bibliometrix; Table S5: Most relevant countries by corresponding author bibliometrix; Table S6: Source Prod over Time bibliometrix; Table S7: Word cloud plot synonyms; Table S8: Word cloud plot words excluded; Table S9: Word frequency distribution; Table S10: VOSviewer thesaurus file.
